# Translational Learnings in the Development of Chemo-Immunotherapy Combination to Bypass the Cold Tumor Microenvironment in Pancreatic Ductal Adenocarcinoma

**DOI:** 10.3389/fonc.2022.835502

**Published:** 2022-05-18

**Authors:** Hélène Kaplon

**Affiliations:** Institut de Recherches Internationales Servier, Translational Medicine Department, Servier, Suresnes, France

**Keywords:** pancreatic ductal adenocarcinoma, schedule, immune checkpoint inhibitor, chemotherapy, metronomic dose

## Abstract

Pancreatic ductal adenocarcinoma (PDAC) is among the most lethal cancers, with a 5-year relative survival rate of 5%. The desmoplastic stroma found in the tumor microenvironment of PDAC is suggested to be partly responsible for the resistance to most therapeutic strategies. This review outlines the clinical results obtained with an immune checkpoint inhibitor in PDAC and discusses the rationale to use a combination of chemotherapy and immune checkpoint therapy. Moreover, essential parameters to take into account in designing an efficient combination have been highlighted.

## 1 Introduction

Pancreatic ductal adenocarcinoma (PDAC) represents 90% of all pancreatic malignancies. PDAC is currently the fourth most common cancer worldwide with the worst 5-year overall survival (OS) rate of 5% across many solid tumors (1, 2). The PDAC incidence is higher in Western countries than in Asia and Africa and is expected to rise over the coming years due to lifestyle, longer lifespan, and public health problems such as obesity and diabetes (3). In 2030, PDAC would be the second leading cause of cancer-related deaths in the United States (US) (4).

At the time of diagnosis, 80% of newly diagnosed patients present with locally advanced or metastatic disease while only about 20% of PDAC patients are candidates for surgical resection (4). For patients with advanced diseases, adjuvant cytotoxic chemotherapy with drugs such as FOLFIRINOX (5-fluorouracil, leucovorin, irinotecan, oxaliplatin) or nab-paclitaxel and gemcitabine is the current treatment option. Indeed, in a phase 2/3 study (NCT00112658), the FOLFIRINOX combination increased the median OS (11.1 months) and median progression free-survival (PFS) (6.4 months) compared to gemcitabine monotherapy (median OS 6.8 months and median PFS 3.3 months) but was associated with an increase in side effects. This regimen is therefore an option for the treatment of metastatic pancreatic cancer with a good performance status (5). Recently, the combination of nab-paclitaxel and gemcitabine has shown to have superior efficacy in terms of OS (8.5 months), PFS (5.5 months), and overall response rate (ORR) compared with gemcitabine monotherapy (with median OS of 6.7 months and median PFS of 3.7 months) in the MPACT Phase 3 study (NCT00844649) (6). Despite these improvements, medians of survival remain insufficient, which show that efforts must continue to provide new strategies to patients.

In recent years, the discovery of immune checkpoints (ICP) has revolutionized immuno-oncology treatments. It has been shown that tumors develop escape mechanisms to avoid recognition by the immune system by expressing ligands such as PD-L1 which binds co-inhibitory receptors like PD-1. By preventing this interaction, ICP inhibitors restore the activation of the immune system which translates into clinical benefit for about 13% patients in many solid tumors (7). Furthermore, biomarker studies have now described the association between high expression of PD-L1 (8) and/or high microsatellite instability (MSI-H) (9) and the response to these treatments.

The study of the PDAC tumor microenvironment (TME) has highlighted several factors which suggest that immunotherapy could have a clinical impact in this cancer type. First, the presence of an immune infiltrate correlated with the prognosis of patients, suggesting the presence of preexisting antitumor immune responses (10–12). Furthermore, in a study, 1/3 of pancreatic tumors has an immune infiltrate similar to that of melanoma supporting the notion that PDAC is a heterogeneous group of tumors and some patients harbor immunogenic tumors (13). Second, the characterization of the tumor microenvironment has highlighted that the expressions of ICP such as CTLA-4, PD-L1, LAG-3, and TIM-3 are associated with poor survival in PDAC tumors (13–15). Finally, reports have shown the presence of tertiary lymphoid structures (TLS), mostly located in the tumor periphery of PDAC tissues. In accordance with their favorable prognosis role in many solid tumors (16), PDAC tumors harboring TLS are enriched with IgG1 memory B cells and memory CD4+ T-cells and a higher expression of Th1- and Th17-related genes (17, 18) combined with a lower infiltration of immunosuppressive cells. Moreover, these TLS are associated with longer survival for patients in PDAC (17). Recently, numerous papers have highlighted the predictive role of these TLS in the response to ICP inhibitors in solid tumors (19) including melanoma (20), sarcoma (21). These findings suggest that PDAC tumors, or at least some of them, could be responsive to ICP inhibitors.

Despite the presence of biological factors that may suggest a potential response to single-agent ICP inhibitors, PDAC tumors do not respond to these immunotherapies. Indeed, although ipilimumab increased the survival of melanoma patients, this anti-CTLA-4 antibody did not meet its primary endpoint in a phase 2 clinical trial for advanced pancreatic cancer patients (22). The combination of two ICP inhibitors (anti-CTLA-4 + anti-PD-L1) remains ineffective for PDAC patients (23). Multiple hypotheses have arisen to explain this lack of response in PDAC. Understanding these resistance factors is a key element in defining new therapeutic strategies and improving responses to the ICP inhibitors of these cold tumors, also called immunological deserts.

## 2 Resistance Factors to Immune Checkpoint Therapy in PDAC

### 2.1 Immune Cell Content

It has been suggested that the TME mediated the suppression of T-cell priming and function in PDAC, thus contributing to resistance to these treatments. Observation of a high density of immunosuppressive cells in the pancreatic intraepithelial neoplasia (PanIN) (24) combined with a dysfunctional T-cell phenotype suggests an impairment of T-cell mediated antitumor responses from the early stages.

It has been shown that tumor-associated macrophages (TAM) accumulate in the stroma of the PDAC TME tumor microenvironment *via* CCL2 (25) secreted by cancer cells. In the tumor, multiple cells including cancer-associated fibroblasts (CAF), regulatory T-cells (Treg), and Th2 cells promote the TAM polarization toward the M2 phenotype (26). Accordingly, many studies revealed that a high density of TAM is associated with poor survival (27, 28), supporting their involvement in the tumor development and progression. TAM can also contribute to T-cell exclusion from tumor islets (29). These conclusions are in line with observations made in mouse models showing that the depletion of TAM not only impaired PDAC cell proliferation (30) but also induced T-cell recruitment within the tumor bed (31) and restored the antitumor activity of T-cells (32).

A study analyzing the Treg contribution in PDAC supports the notion that myeloid immunosuppressive cells are the most contributor to the tumor progression in PDAC. Interestingly, Treg depletion has failed to inhibit tumor growth due to the establishment of compensatory mechanisms such as an increase of myeloid cells and CAF reprogramming (33). CAFs are known to generate dense fibrosis or desmoplasia within and around the tumor. This desmoplastic stroma is composed of pancreatic stellate cells (PSC), CAF, and extracellular matrix (ECM) components and represents up to 80% of the tumor volume which is a key feature of the PDAC TME. Whether this desmoplastic stroma prevents immune infiltration is still a matter of debate. Indeed, *ex vivo* models support the notion that excessive collagen deposition impedes T-cell entry into the TME as the collagen degradation has increased T-cell infiltrates (34). However, the study of the spatial relationships between T-cell subpopulations and cancer cells has shown that the desmoplastic stroma has no impact on T-cell infiltration (35). Surprisingly, the depletion of αSMA+ CAF in mice could reduces desmoplasia but enhances hypoxia and epithelial-to-mesenchymal transition, promotes tumor progression, and is associated with reduced survival (36). These findings suggest that desmoplastic stroma is also involved in the control of tumor growth.

### 2.2 Low Immunogenicity

Immunogenicity is defined as the ability of antigens to induce immune responses. In the TME, some of the somatic mutations occurring in cancer cells generate neoepitopes that can be loaded on to major histocompatibility complex (MHC) molecules and eventually activate specific T-cells. It is likely that tumors with a high number of somatic mutations have statistically more immunogenic neoantigens, that is why the tumor mutational burden (TMB) representing the number of somatic mutations per mega base in the genome of a cancer cell is currently used as an estimation of antigen load in a tumor. However, a recent study has demonstrated that TMB is not a predictive biomarker of ICP-inhibitor therapy for all solid tumors including PDAC, in which CD8 T-cell infiltration is not associated with neoantigen load (37). In the same line, data from long-term survivors in PDAC have shown that neoantigen quality rather than quantity conferred higher tumor immunogenicity (38).

Unlike some other solid tumors such as melanoma and lung cancer with high TMB (>10 mutations/Mb), PDAC tumors exhibit a low median TMB (2.7 mutations/Mb), which may explain the ineffectiveness of ICP inhibitors (39). Impairment of mismatch repair (MMR) leads to an increase in the number of mutations and neoantigens conferring microsatellite instability status. As observed with pembrolizumab, MSI-high (MSI-H) tumors are more likely to respond to ICP inhibitor therapy. However, about 1% of PDAC tumors harbor MSI-status, suggesting that only a small number of PDAC patients may benefit from pembrolizumab (40, 41).

Taken together, these data clearly demonstrate that many immunological parameters interfere with the clinical activity of immunotherapy in PDAC. The current challenge is therefore to overcome the barriers of this “cold” TME tumor microenvironment and to use certain strategies improving tumor immunogenicity in order to convert them into “hot” tumors. In this review, we discuss the use of the chemo-immunotherapy combination to sensitize pancreatic tumors to immune therapy and modalities to take into account in designing efficient combinatorial approaches.

## 3 Immune Checkpoint-Based Chemoimmunotherapy Combination

### 3.1 Rationale of the Combinatorial Strategy

As observed in previous clinical studies, the targeting the TME using single-agent ICP inhibitors is not enough for PDAC patients. Although conventional chemotherapies have immunosuppressive effects as seen with lymphopenia and neutropenia in treated patients, these chemotherapeutic agents also have immunostimulatory properties that can be exploited to improve the survival of PDAC patients.

**Figure 1:**
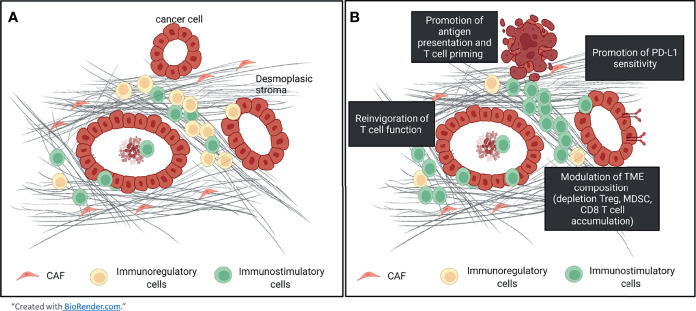
The tumor microenvironment of pancreatic ductal adenocarcinoma and impact of the chemo-immunotherapy. **(A)** The tumor microenvironment of the pancreas is highly infiltrated by immunosuppressive cells (MDSC, Treg, M2 macrophages). **(B)** Chemotherapy promotes T cells recruitment and their priming which could counterbalance the ratio immunosuppressive / effector cells within the TME while ICP inhibitor reinvigorates exhausted T cells. Moreover, an upregulation of PD-L1 expression has been observed and may increase the sensitivity to PD-(L)1 inhibitors.

For example, an *in vivo* study has demonstrated that 5-FU drug depletes immunosuppressive cells (MDSC, Treg) while increasing IFN-γ production by tumor-infiltrating CD8 T-cells and is associated with antitumor immune responses (42). It appears that many chemotherapeutic agents could bypass the permissive TME in favor of antitumor immunity by using different mechanisms such as the depletion of immunosuppressive populations combined with a recruitment of cytotoxic T-cells within the TME, an induction of DC maturation (43) with an increased antigen presentation ability, as well as an upregulation of MHC-I (44) and PD-L1 expression on tumors cells (45). the immunological effects of chemotherapeutic drugs used in the clinical management of PDAC tumors are summarized in the **Table 1**. By promoting the generation of neoantigens, chemotherapy enables T-cell recruitment and priming in addition to cell depletion of immunosuppressive cells while ICP inhibitor stimulates exhausted T cells **(** **Figure 1** **)**. This synergistic effect has been demonstrated in several preclinical (122) and clinical (123, 124) studies where the combination lengthened the median OS with an acceptable safety profile (123).

**Table 1:** Immunomodulatory effects of chemotherapeutic agents used for the treatment of pancreatic ductal adenocarcinoma.

Chemotherapy class	Molecule	Immune-related effects	References
Anti-metabolite	5-FU	Several cycles decrease CD8 T-cell proliferation, cytotoxicity, and IFN-γ secretion of spleen cells (M)	(46)
		Increased IFN-γ production by tumor-specific CD8 T-cells infiltrating the tumor (M)	(42)
		Decreased number of circulating B cells (M)	(47)
		Depletion of splenic B cells while lymph node B cells are not affected (M)	(48)
		Depletion MDSC in the spleen and in the tumor bed (M)	(42)
		Increased circulating Tregs (M)	(47)
		Increase of TAM and Treg infiltration in gastric cancer (H)	(49)
		Increased B7-H6 expression on tumor cells (M)	(50)
		Upregulation of PD-L1 in gastric cancer (H)	(51–53)
	Gemcitabine	Decrease of ICOS+ CD8 T-cells frequency in draining lymph nodes with a significant decrease on the level of intratumoral Ki-67-expressing cells (M)	(54)
		Decrease in the absolute number of intratumoral CD8 T-cells (M)	(54)
		Decrease of IFN-γ-producing CD4 and CD8 T-cells in the tumor (M)	(55)
		Increased ratio of T conv/Treg in the tumor (M)	(56)
		Decrease of circulating Treg levels in blood from pancreatic cancer and increase of the Teff/Treg ratio (H)	(57)
		Decrease of circulating MDSC associated with an increased peripheral T- and NK-cell proliferation (H)	(58)
		Decrease of Treg levels in blood (M)	(59)
		Promotion of TAM accumulation and CSF1, CCL2 upregulation (M)	(60)
		Increase of monocytes and CD11c+ dendritic cells in pancreatic cancer (H)	(61)
		Depletion of MDSC, macrophages and eosinophils in the tumor (M)	(62)
		Switch towards antitumor macrophage profile (H)	(63)
		Upregulation of M2-polarized macrophage markers such as Arg1 and TGF-β (M)	(64)
		Decreased suppressive TAM frequency in the tumor (M)	(65)
		Decrease of MDSCs, Tregs, and macrophages in the tumor (M)	(66)
		Reduction of IFN-γ secretion from CD8+ T cells and inhibition of T-cell activation (H)	(67)
		Depletion of MDSC in the spleen (M) (65) and tumor (M)	(68)
		Decrease of TGF-β and induction of M2 recruitment in the tumor (M)	(69)
		Decrease of peripheral memory T-cells, after several infusions (H)	(70)
		Decreased Antibody titers (M)	(71)
		Upregulation of CD47, CD73, and PDL1 at mRNA levels (H, M)	(72)
		PD-L1 upregulation in cell lines at both mRNA and protein levels (M, H)	(51, 73)
		Upregulation of PD-L1, CD47 and MHC-I on cell lines (H)	(67)
		Increased MHC-I expression on tumor cells in ovarian cancer (M, H)	(74)
		Upregulation of PD-L1 on myeloid cells in pancreatic cancer (H)	(57)
		ICOS, CD28 and HLA-DR expression on circulating CD4 and CD8 T cells and NK cells in mesothelioma (H)	(58)
		Upregulation of NKG2D ligands (MICA and MICB) and MHC-I expression on tumor cells (H)	(75, 76)
	Capecitabine	Increase of CD4+, CD8+ central memory T cells, NK cells (H)	(77)
		Depletion of circulating MDSC in glioblastoma (H)	(77)
		Decrease of CTLA-4 expression in lymphocytes while no alteration of TIM3 and LAG3 expression was observed (H)	(77)
Platinum	Cisplatin	Increase of CD8 T-cell infiltrate into tumor tissues (M)	(78)
		Increase of monocytes in the tumor (M)	(79)
		Increase in T-cell and monocyte/macrophage activation markers (CD62L, CD301) (M)	(79)
		Decreased frequency of ICOS+ CD4+ or CD8+ T cells in the draining lymph node (M)	(54)
		Downregulation of CD80, CD86, MHC-I, MHC-II expression on DC combined with an increased IL-10 production (M)	(80)
		Decrease of IL10, IL6, and VEGF (M)	(79)
		Decrease of the accumulation of peripheral myeloid cells (M)	(81)
		Depletion of MDSC in tumor-draining lymph node (M)	(82)
		Tumor-derived MDSC downregulated Gr1 expression and upregulated CD40 phenotype (M)	(82)
		Reduction of Breg frequency and decrease of adenosine production in HNSCC (H)	(83)
		Decrease of PD-L1 and PD-L2 expression in DC (H)	(84)
		Increased expression of MHC-I and PD-L1 (M, H)	(85–87) (88)
		Upregulation of PD-L1 expression in HNSCC cell line (H)	(89, 90)
		Increased expression of CD70, CD80, and CD86 on antigen presenting cells (APC) (M)	(91)
		Induction of MHC-I expression in colon cell line (H)	(92)
		Upregulation of MICA/B expression at protein level in NSCLC (H)	(93)
		Upregulation of MHC-I and PD-L1 (M)	(85)
	Oxaliplatin	Increase of CD4 and CD8+ T-cell infiltrate in the spleen and increase of activated T-cells and TNFα expression (M)	(94)
		Depletion of splenic B cells (M)	(94)
		Increased number of PD1+ CD8+ T-cells in blood circulation (M)	(95)
		Increased expression of T-cell chemoattractant CXCL9, CXCL10, and CCL5 in tumor cells (M)	(95)
		Increased immune cell infiltration in tumor including CD8 T-cells (M)	(96)
		Induction of CD8 T-cell (not CD4) recruitment into tumors (M)	(97)
		Decreased frequency of ICOS+ CD4 and CD8 T-cells in lymph node (M)	(54)
		Decrease in IFN-γ+ CD8 T-cells in the tumor (M)	(54)
		Increased number of CD4 and CD8 T-cells in the tumor (M)	(98)
		Decreased frequency of ICOS+ CD4 or CD8 T-cells in draining lymph node (M)	(54)
		Increased Treg infiltration in the spleen (M)	(94)
		Decrease of macrophages and DC numbers in lymph node (M)	(99)
		Increased infiltration of IgA+ PD-L1+ IL10+ plasma cells in the tumor (M)	(96)
		Depletion of MDSC in the tumor and promotion of their differentiation into mature cells such as macrophages or DC (M)	(98)
		Decrease of Treg in the tumor (M)	(98)
		Decrease of PD-L1 and PD-L2 expression in DC (H)	(84)
		Increased expression of MHC-I and PD-L1 (M, H)	(85)
		High level of PD1 and TIM3 expression on CD8 in the tumor (M)	(95)
		Upregulation of PD-L1 expression on tumor cells (M)	(95)
	Carboplatin	Increase of CD4 and CD8 T-cells in the tumor (M)	(100)
		Increase of CD8 T-cell infiltrate in the tumor (M)	(101)
		Increase in IFN-γ+ CD8 T-cells in the tumor (M)	(54)
		Differentiation of MDSC and activation of the IL13/33 axis (M)	(102)
		Decrease of Treg and MDSC in the tumor (M)	(100)
		Promotion of Treg accumulation via IL10 secreted by MDSC in the tumor (M)	(102)
		Upregulation of CD47, CD73, and PDL1 at mRNA level (H, M)	(72)
		Increased PD-L1 expression on tumor cells in ovarian cancer (H)	(103)
		Decreased PD-L1 and PD-L2 expression in DC (H)	(84)
Taxanes	Docetaxel	Upregulation of CXCL11 and enhancement of CD8 T-cell recruitment	(104)
		Promotion of M1 polarization (H) and activation	(105)
		Induction of IL-8 and IL-1β secretion by monocytes (H)	(105)
		Accumulation of TAM (M)	(106)
		Decrease of MDSC proportion in the spleen and induction MDSC polarization towards an M1 like phenotype (M)	(107)
		Inhibition of PBMC proliferation and apoptosis of activated PBMC (H)	(108)
		Treg depletion after several doses (H)	(109)
		Decreased PD-1 expression on T cells	(110)
		Upregulation of PD-L1 expression in cells (M)	(111)
	Paclitaxel	Increase of CD8 T-cell infiltrate in ovarian tumor (M, H)	(74)
		Increase of the priming of CD8 T cells (M)	(112)
		Induction of M1 phenotype (M, H)	(113, 114)
		Induction of IL12 production by macrophages (M)	(115)
		Upregulation of maturation markers (MHC-II, CD86) on DC (M)	(43)
		Induction of GM-CSF mRNA production in cells	(116)
		Decreased MDSC infiltrate associated with an inhibition of TNF and S100A9 expression in the tumor (M)	(117)
		Decrease of Treg numbers in the tumor (M)	(112)
		Upregulation of CD47, CD73, and PDL1 at mRNA level (H, M)	(72)
		Increased MHC-I expression on tumor cells in ovarian cancer (M, H)	(74)
		Upregulation of PD-L1 expression on tumor cells (M,H)	(51, 74, 103) (111)
		Upregulation of PD-L1 and MHC-I on tumor cells (M)	(118)
Topoisomerase I inhibitor	Irinotecan	Increase of CD8 T-cells in tumor (M)	(119)
		Decrease of Treg (M) in the tumor and lymph node (M)	(119, 120)
		Increased PDL1 and MHC-I expression on tumor cells (M)	(119, 120)
		Increased MHC-I expression on tumor cells (H)	(121)

Data were collected in mice (M) or human (H) cell line, biopsy or in vitro.

However, this clinical benefit is not obvious, as demonstrated by the negative results of the phase 1b trial (NCT01473940) combining gemcitabine + ipilimumab in late-stage PDAC patients (125). These clinical observations suggest that some parameters (chemotherapeutic agents, dose regimen, schedule of administration) are not optimal yet and should be assessed to design efficient combinatorial strategies.

### 3.2 Modalities for This Combinatorial Strategy

#### 3.2.1 Chemotherapeutic Agents

The immunological effects of some chemotherapeutic agents could be deleterious in some settings. For example, in PDAC, gemcitabine promotes the accumulation of macrophages and their polarization toward the pro-tumor M2 phenotype (64, 69). In response to some chemotherapeutic agents, TAM (126) and CAF (127) secrete VEGF-A, VEGF-C and other pro-angiogenic factors (128). In mice, gemcitabine increases the synthesis of some chemokines and TGF-β signals leading to gemcitabine resistance (129). However, this drug is currently part of the standard of care, suggesting that these pro-tumor effects must be counterbalanced by the immunostimulatory effects of the drug. Other examples of the controversial effects of chemotherapeutic agents are reported in **Table 1** with the accumulation of regulatory and immunosuppressive cells after treatment in mice and in humans. For example, oxaliplatin or carboplatin favors Treg or MDSC infiltration which is related to chemoresistance (102).

These findings suggest that a better understanding of these immune-mediated effects of chemotherapy is required to find the most promising combinatorial strategies. Moreover, other parameters such as the dose regimen, schedule of administration, drug treatment schedule, and tolerability profile are not clearly understood yet and are also crucial to achieving clinical success.

#### 3.2.2 Dose Regimen: Standard or Metronomic?

Conventional chemotherapeutic agents are commonly used at the maximum tolerated dose (MTD) which represents the highest dose of the drug acceptable for the patient in terms of toxicity. By their mechanism of action, these treatments exert strong cytotoxicity on proliferating hematopoietic cells, including immune cells, resulting in profound myelosuppression and a risk of infections. These toxicities require administration interspersed with drug-free periods to restore hematopoiesis, periods during which chemoresistant clones may emerge. In contrast, metronomic doses of chemotherapy, i.e., administration of chemotherapeutic agents at low dose but frequently, could bypass deleterious effects (130) of conventional chemotherapy with higher efficacy to control the disease (131).

The antitumor effects of the metronomic vs. standard dose of chemotherapy were assessed in several PDAC mouse models especially for cyclophosphamide and gemcitabine agents (132–134). The studies showed that the reduction in tumor growth was equivalent in both regimens (132, 133), but a metronomic administration of gemcitabine induces anti-angiogenic effects as observed by the induction of thrombospodin-1 (TSP-1), an angiogenic inhibitor factor (132). Similarly, Cham et al. demonstrated the decrease of pro-angiogenic factors such as such EGF, IL-1α, IL-8, ICAM-1, and VCAM-1 in the tumor after a metronomic dose of gemcitabine as well as decreased hypoxia (133, 134). In addition to this anti-angiogenic activity, a low dose of gemcitabine could also impact the immune cell content. In an orthotopic model of PDAC, low-dose gemcitabine depletes Treg, thus inducing a concomitant increase of conventional T-cell percentages but have no impact on the frequency of MDSC (56).

A metronomic dose of chemotherapy is also effective for PDAC patients while being less toxic. Indeed, the combination of a low dose of nab-paclitaxel (60 mg/m²) + oxaliplatin (50 mg/m²) plus a continuous infusion of 5-FU and bevacizumab (anti-VEGF) was effective with an ORR of 49% and a disease control rate (DCR) of 81%. Surprisingly, 82% of patients were still alive beyond 1 year (135).

This beneficial effect at a metronomic dose might be mediated by depletion of immunosuppressive populations such as Treg (136, 137), promotion of DC maturation, enhancement of T-cell-mediated antitumor immunity (138) and/or anti-angiogenic properties as initially described with cyclophosphamide (139, 140). However, although there is a certain amount of preclinical evidence demonstrating the positive impact of low-dose chemotherapy on the TME, this benefit is not well documented in clinic. Indeed, few ongoing clinical trials ongoing clinical trials in combination with an ICP inhibitor use this dose regimen (**Table 2**).

**Table 2:** Ongoing clinical trials in PDAC using chemotherapy in combination with immune checkpoint with associated doses and schedules.

Clinical trials	ICP	Agents	Phase	Final	Chemotherapy dose	Treatment schedule
NCT04827953	CTLA-4 (zalifrelimab)	Nab-paclitaxel Gemcitabine Hedgehog pathway inhibitor	I/II	June 2023	Standard	NR
NCT03496662	PD-1 (nivolumab)	Nab-paclitaxel Gemcitabine CCR2/5 inhibitor	I/II	Oct 2024	Standard	Concomitant
NCT04753879	PD-1 (pembrolizumab)	Nab-paclitaxel Gemcitabine Cisplatin Irinotecan Capecitabine Olaparib	II	Dec 2029	Low dose	Chemotherapy followed by ICP inhibitor
NCT04581343	PD-1 (spartalizumab)	Nab-paclitaxel Gemcitabine Anti-ILb (canakinumab)	IB	June 2022	Standard	Concomitant
NCT04390763	PD-1 (spartalizumab)	Nab-paclitaxel Gemcitabine Anti-TGFb	II	May 2025	Standard	NR
NCT04083599	PD-1 (pembrolizumab)	Nab-paclitaxel Gemcitabine CD40×4-1BB agonistic Ab	I/II	Sept 2025	NR	Concomitant followed by ICP inhibitor + agonistic antibody
NCT03611556	PD-L1 (durvalumab)	Nab-paclitaxel Gemcitabine mFOLFOX (oxaliplatin, leucovorin, 5-FU) Anti-CD73	I/II	Dec 2022	NR	NR
NCT03193190	PD-L1 (atezolizumab)	Nab-paclitaxel Gemcitabine Anticancer agents	I/II	June 2024	Standard	Concomitant
NCT02754726	PD-1 (nivolumab)	Nab-paclitaxel Gemcitabine Cisplatin Paricalcitol	II	June 2023	Standard	Concomitant
NCT05031494 NCT04481009	PD-1 (toripalimab)	Nab-paclitaxel Gemcitabine YH003	II	Dec 2023 March 2023	NR	NR
NCT04247165	PD-1 (nivolumab) CTLA-4 (ipilimumab)	Nab-paclitaxel Gemcitabine Radiation	I/II	Feb 2024	Low dose	Concomitant
NCT04787991	PD-1 (nivolumab) CTLA-4 (Ipilimumab)	Nab-paclitaxel Gemcitabine Hydroxychloroquine	I	Oct 2023	Standard	NR
NCT04543071	PD-1 (cemiplimab)	Nab-paclitaxel Gemcitabine Motixafortide	II	August 2025	Standard	NR

NR, non reported.

#### 3.2.3 The Administration Schedule : Concomitant or Sequenced Regimen?

To date, several clinical trials investigating the efficacy and safety of concomitant chemoimmunotherapy are ongoing. However, according to the scientific rationale, the best treatment sequence would be the administration of chemotherapy first, which sensitizes the TME by releasing neoantigens and promoting T-cell priming, followed by ICP inhibitor therapy which may sustain T-cell-mediated antitumor activity.

However, in a preclinical pancreatic model, the concomitant administration of chemotherapy and PD-L1 blockade results in complete responses compared to the sequenced administration of chemotherapy followed by an anti-PD-L1 therapy (15). The same result was reported in a mesothelioma mouse model where the synergistic effect of the combination was only observed when both drugs were administered simultaneously (141). Conversely, in a phase 2 study, the efficacy of sequential administration versus concomitant administration of chemoimmunotherapy combination has been tested for the treatment of metastatic melanoma. In this case, the sequential use of ipilimumab followed by chemotherapy confers a PFS benefit (142). This effect was not seen in PDAC, where sequential administration of chemotherapy and immunotherapy is not associated with an improved OS (143). As with current clinical guidelines, most clinical trials ongoing in PDAC (**Table 2**) deliver chemotherapy concomitantly with immunotherapy, but data are lacking to support the current treatment schedule suggesting a window for improvement. These findings demonstrate that a scheduled regimen may require to be adapted according to the tumor type and therapeutic agents.

## 4 Conclusion

Pancreatic ductal adenocarcinoma is one of the most aggressive and deadly cancers. With the lowest 5-year survival rate among solid tumors, the medical need is high and requires a great deal of effort from researchers to find therapeutic strategies that are more effective than current chemotherapies. Unfortunately, despite the arrival of breakthrough ICP inhibitor therapy, survival for PDAC patients has not improved significantly. The study of the microenvironment has highlighted that low immunogenicity of PDAC tumors limits the effectiveness of current treatments. One strategy to circumvent these barriers is the use of chemotherapy to sensitize this permissive microenvironment in combination with ICP inhibitors. The efficacy of this combinatorial strategy has been reported in multiple tumor types including PDAC. However, the observed clinical benefit of these combinations is not universal and seems to be dependent on several parameters. In this review, we have shown that some chemotherapeutic agents have pro- and antitumor effects. However, the molecular characteristics of the modulations induced by these treatments are not sufficiently established and could be informative to designing more efficient combination strategies. These immunological effects can be modulated by the type, the dose regimen and the administration schedule. Accumulating evidence has demonstrated equivalent antitumor effects between low-dose chemotherapy and standard dose chemotherapy; these studies reported additional activities of low-dose chemotherapy such as inhibiting hypoxia and reducing angiogenesis. Despite this rationale, few combinations under investigation in clinical trials use. This dose regimen which could improve combination tolerability. Regarding the administration schedule regimen, to date, many clinical trials are testing the efficacy of chemotherapy administered concomitantly with ICP inhibitor therapy, mainly at MTD levels. Preclinical and clinical data obtained in studying the impact of this parameter on antitumor response are quite confusing. This administration schedule is likely to depend on the dose as well as the therapeutic agent chosen. Currently, data obtained in studying the combination of these parameters are lacking. Future research should therefore explore the impact of these treatment modalities on preclinical models and subsequently in clinical trials to guide the development of appropriate synergistic combinations.

Finally, the heterogeneity of PDAC patients is also a crucial parameter to consider. Some of PDAC subtypes are more immunogenic with a greater chance to response to ICP inhibitor therapy, while others are an immune desert. As 80% of PDAC tumors are unresectable at diagnosis, which hinders knowledge of the disease, the development of omic? Technologies will help leverage and collect as much biomarker data as possible from tumor samples in the clinic to gain a deeper understanding of the TME and monitor pharmacodynamic biomarkers to optimize combination parameters.

## Author Contributions

The author confirms being the sole contributor of this work and has approved it for publication.

## Conflict of Interest

Author HK was employed by Servier.

## Publisher’s Note

All claims expressed in this article are solely those of the authors and do not necessarily represent those of their affiliated organizations, or those of the publisher, the editors and the reviewers. Any product that may be evaluated in this article, or claim that may be made by its manufacturer, is not guaranteed or endorsed by the publisher.

## Abbreviations

CAF, cancer-associated fibroblast; DAMPS, damage-associated molecular pattern; DC, dendritic cells; DCR, durable clinical response; ECM, extracellular matrix; HIF-a, hypoxia-inducible factor 1; ICP, immune checkpoint; MDSC, myeloid-derived suppressor cells; MHC, major histocompatibility complex; MMR, mismatch repair; MTD, maximum tolerated dose; NR, not reported; ORR, overall response rate; OS, overall survival; PanIN, pancreatic intraepithelial neoplasia; PDAC, pancreatic ductal adenocarcinoma; PFS, progression-free survival; PRR, pattern recognition receptors; PSC, pancreatic stellate cells; TAM, tumor-associated macrophages; TLR, toll-like receptors; TLS, tertiary lymphoid structure; TMB, tumor mutational burden; TME, tumor microenvironment; Treg, regulatory T cells; TSP-1 thrombospondin-1; US, United States.
